# SARS-CoV-2 surveillance and vaccine effectiveness among dental healthcare workers during the omicron surge

**DOI:** 10.1038/s41598-026-50216-8

**Published:** 2026-05-05

**Authors:** Holly Shoemaker, Jeri L. Bullock, Kristina Stratford, Melodie L. Weller, Wendy L. Hobson, Jeanmarie Mayer, Yue Zhang, Vanessa Stevens, Stacey E. Swilling, Matthew Samore

**Affiliations:** 1 Department of Population Health Sciences, Spencer Fox Eccles School of Medicine, Salt Lake City, UT USA; 2 Veterans’ Affairs Salt Lake City Health Care System, IDEAS Center of Innovation, Salt Lake City, UT USA; 3 School of Dentistry, University of Utah, Salt Lake City, UT USA; 4 Division of Epidemiology, Department of Internal Medicine, Spencer Fox Eccles School of Medicine, Salt Lake City, UT USA; 5 Department of Pediatrics, Spencer Fox Eccles School of Medicine, Salt Lake City, UT USA; 6 University of Utah, 50 N Medical Dr, Salt Lake City, UT 84132 USA

**Keywords:** COVID-19, SARS-CoV-2, Vaccine effectiveness, Dental healthcare workers, PCR surveillance, Survival analysis, Dental education, Infection control in dentistry, Viral infection, Epidemiology

## Abstract

Dental students and practitioners may have an increased risk of COVID-19 infection due to frequent aerosol-generating procedures (AGP) and close patient contact. We examined the role of vaccination status, work role, and AGP frequency on positive SARS-CoV-2 PCR surveillance test using a Cox proportional hazards regression and weighted for dropout. A total of 410 dental health workers (200 students, 104 faculty, and 106 staff) had 8,270 screening tests performed between May 2020 and February 2022, with 158 positive tests; 60 (38%) occurred in January 2022. Omicron had a significant impact on vaccination effectiveness. Vaccine effectiveness within < 4 months was 91% (HR: 0.09,95% CI: 0.02–0.40) prior to Omicron, which decreased after its emergence. Work role was not associated with risk of positive test. Reported AGP frequency was also not associated with positive test risk; however, these analyses were limited to a subset of participants and should be considered exploratory. More than a third of all positive tests occurred during one month of Omicron. We found a high vaccine effectiveness prior to the Omicron surge, which decreased after the emergence of Omicron. Our results support encouraging dental healthcare workers (DCWs) to maintain up-to-date vaccination and continue engaging in preventive measures.

**Supplementary Information:**

The online version contains supplementary material available at 10.1038/s41598-026-50216-8.

## Introduction

During the COVID-19 pandemic, dental practitioners and dental education institutions faced significant challenges regarding safety in the clinical setting^1^. On March 16th, 2020, the American Dental Association called on dentists to postpone elective dental procedures to mitigate the spread of severe acute respiratory syndrome coronavirus type 2 (SARS-CoV-2), which causes COVID-19^2^. SARS-CoV-2 is highly transmissible, does not require close contact or fomite contact for transmission, and transmission can occur over long distances (> 2 m) through respiratory droplets and aerosols^3^. Aerosol-generating procedures (AGPs) are routine in medical and dental settings and produce airborne particles from saliva, respiratory secretions, or other fluids that can remain suspended in the air^4–6^. Examples of AGPs in dentistry with high contamination risk may include ultrasonic scaling, air-water syringe, air polishing, and extractions using motorized handpieces^7^. These procedures, combined with prolonged close contact with patients, may increase the risk of SARS-CoV-2 exposure and transmission to dental healthcare workers (DCWs). Furthermore, exposure levels can vary by work role within dental practice, as individuals may have differing degrees of patient interaction and involvement in AGPs^4^. Workplace exposure to aerosols and infectious material can be mitigated through multiple strategies, including the use of personal protective equipment (PPE) such as KN95 or N95 respirators, environmental controls, vaccination, and regular screening of personnel and patients^8–11^.

The rate of COVID-19 among dental professionals has been observed to be low^12,13^, potentially due to preventive measures almost universally reported in studies of dental practitioners^10^. However, little evidence is available to understand whether the preventive measures impacted transmission in the dental care setting and how transmission and risk varied based on the frequency of AGP exposure or how risk varied among dental care worker roles, including non-clinical staff.

To mitigate the risk of COVID-19 transmission upon re-opening clinical operations, the University of Utah School of Dentistry (UUSOD) implemented environmental control measures and screening of patients with polymerase chain reaction (PCR) testing. In May 2020, the UUSOD began regular asymptomatic surveillance polymerase chain reaction (PCR) testing of patient-facing faculty, staff, residents, and students. Vaccination of DCWs was encouraged when the COVID-19 vaccine first became available in December 2020. The emergence of the Omicron variant represented a large shift in the course of the pandemic. Omicron BA.2 and subsequent variants demonstrated increased transmissibility compared to previous variants^14^, and prior Omicron variants had already shown increased ability to evade the immune system^15^.

This study aims to evaluate the effectiveness of the prevention measures taken by the UUSOD during this period. The primary outcome was to determine if the Omicron variant impacted vaccine effect on prevention of infection among DCWs. A secondary outcome was to determine if work role (faculty, staff, or student) or self-reported AGP influenced the likelihood of positive SARS-CoV-2 surveillance tests. We utilized data from a cohort of dental education faculty, residents, students, and staff working in the dental care setting who underwent regular PCR surveillance screening for COVID-19. Time since most recent vaccine, the presence of the Omicron variant, work role, and self-reported frequency of AGP were hypothesized to be associated with time to positive surveillance test. Understanding the epidemiology of infection within these groups and their relationship to factors, such as vaccine effectiveness and self-report of AGP frequency, can help us understand risks in dental care settings and ensure the workplace safety of all DCWs.

## Methods

### Study design

This study was a prospective longitudinal cohort of DCWs, who were followed over time with regular SARS-CoV-2 surveillance testing. Cross-sectional survey data were incorporated to characterize the self-reported frequency of AGP exposure.

### Ethics

This study was approved by the institutional review board of the University of Utah (IRB_00136660). All experiments were performed in accordance with relevant guidelines and regulations. Informed consent was obtained from all survey participants. Consent was waived for the portion of the study that does not use survey data.

### Setting, participants, & procedures

#### Setting & participants

This study took place at the University of Utah School of Dentistry (UUSOD), an academic dental school in an urban setting, with 200 dental students, 104 faculty and 106 staff. Faculty are defined as licensed dentists providing direct and indirect supervision of clinical procedures, and non-clinical faculty providing didactic lectures and simulation-based education. Staff are defined as non-faculty individuals employed at the UUSOD to support clinical and educational operations. Students are defined as pre-doctorate dental students attending a four-year program at the UUSOD. A formal sample size calculation was not conducted, as this study was based on a defined occupational cohort of DCWs where surveillance testing was required for all in-person working employees during the study period. Survey-based AGP exposure data were obtained from a convenience sample of participants from a larger survey of healthcare professionals.

#### Procedures

##### Prevention measures

A timeline of prevention measures used during the course of this study are listed in Table S1. These included patient pre-appointment testing, increasing spacing between patients at the clinic, adding HEPA filters, reducing the number of aerosolizing procedures, and requiring all clinical providers to use respirators during patient care.

##### Surveillance testing

Starting in May 2020, surveillance testing for SARS-CoV-2 was required for everyone working in the UUSOD clinical building. PCR testing occurred every other week for non-vaccinated DCWs through February 2022, and after May 2021 fully vaccinated DCWs were tested monthly. Everyone was expected to seek additional testing if symptoms or an exposure occurred. Individuals who tested positive completed the appropriate isolation recommended by the CDC^16^ before returning to work or school. DCWs were excused from surveillance testing for 90 days following a positive result.

##### Demographics and survey data

Demographic and vaccination information for participants was self-reported from DCWs who underwent surveillance testing. A subset of participants (*n* = 115) voluntarily opted to complete an additional survey during the study period. This survey collected self-reported information on the frequency of involvement with aerosol-generating procedures (AGP). Because participation was optional, the survey represents a convenience sample that overlapped with the timeframe of the surveillance testing.

#### Exclusion/inclusion criteria

Figure S1 demonstrates the inclusion and exclusion criteria and final analysis cohort. Tests between 05/01/2020 and 03/07/2022 were included. Observations before May 1, 2020 were excluded as surveillance testing was not consistently performed. Dental residents were excluded due to the small group size (*n* = 10) and early loss of follow-up with graduation. Individuals with incomplete data for vaccination status, age, or gender were excluded. For the vaccination analysis, those who reported being vaccinated prior to their first observation (*n* = 3) were excluded, as their time at risk would differ from those who entered the study without being vaccinated. Additionally, individuals missing exposure data for AGP (*n* = 284) were excluded from analyses that use AGP.

#### Definitions

##### Exposure

Vaccination status was defined as one of three categories: unvaccinated or not fully vaccinated, < 4 months since most recent vaccination, and > 4 months since most recent vaccination. Two or more vaccines were considered fully vaccinated, and an individual was not considered vaccinated until 2 weeks after their most recent vaccination.

##### Outcome

PCR testing was reported as positive or negative. Time to test positivity was calculated as the time of study entry (first surveillance test) to the time of the first positive test or the end of the study period, whichever came first. Participants were removed from the dataset after the first positive and not included in the time-to-event analysis. However, repeat-positive tests were included in the descriptive summary table to provide a fuller characterization of the population and events. Nucleic acid amplification testing for SARS-CoV-2 was conducted by ARUP Laboratories, a CLIA-certified laboratory using validated PCR-based methods^17^.

Vaccine effect on infection susceptibility was defined as continuing to test negative on surveillance screening for SARS-CoV-2.

#### Covariates & stratifying variables

AGP exposure was assessed with the question: “How often do you provide care to patients during aerosol-generating procedures? (i.e., intubation, extubation, CPAP, Bi-PAP, open airway suctioning, aerosolized medication, bronchoscopy, upper endoscopy)”. Possible answers included: ‘Frequently (at least once a shift),’ ‘Often (at least once per week),’ ‘Occasionally (at least once per month),’ and ‘Never/Rarely.’ AGP response categories were condensed into ‘Often/Frequently (Once per week or more)’ and ‘Once a month or less.’ As this was a broader survey of healthcare professionals, examples included medical AGPs rather than dental-specific AGPs. Although these examples are not specific to dentistry, participants in this study were exclusively DCWs and assumed to be well-versed in what AGPs would refer to in their practice. As such, responses are interpreted as reflecting relative frequency of self-reported AGP exposure within a dental care context. AGP exposure was assessed at a single time point via survey and treated as a time-invariant covariate. This approach assumes that participants’ relative frequency of involvement in aerosol-generating procedures remained broadly consistent over the study period.

Work role was defined as faculty, staff, and student categories. Work role was included in the analysis both to assess effect and control for potential confounding effects due to group-level differences.

Additional covariates (age and gender) were selected from available data based on clinical significance. Age and gender are both important with regard to health and immune responses, as well as related to cultural factors that influence how individuals interact with one another and transmit disease.

The first cases attributed to Omicron variants were observed early in December in the US^18^, and models estimated that Omicron was the predominant variant by mid-December^18^. We considered observations on and after December 1, 2021 to be part of the Omicron wave when Utah experienced a surge in COVID-19 cases due to the Omicron variant. Although cases occurring between December 1 and December 15, 2021 may reflect a transitional period prior to Omicron predominance, no positive SARS-CoV-2 tests were observed in this cohort during that interval. Therefore, the selected cut-off does not affect classification of observed events. In the remainder of the manuscript, when we refer to “Omicron” or “Omicron wave”, we are referring to any test after this date as defined here.

### Analysis

General descriptive statistics were calculated using counts and percentages. A Kaplan-Meier curve was used to display a descriptive view of the survival (positive COVID-19 test endpoint) of work roles over time. Crude incidence rate (new cases/Person*months) per 1,000 was calculated in the pre and post Omicron time periods.

The risk of a positive surveillance test for each exposure was assessed using a Cox proportional hazards regression with time-dependent covariates (with vaccination being the primary time-dependent covariate). This approach was chosen because it allows for analysis of time-to-event data while accounting for variable follow-up time across participants^19^. We tested for the impact of Omicron on vaccination through an interaction term and calculated vaccine effect by adding and exponentiating the appropriate terms from the model. For calculations that apply to the pre-Omicron time range (reference level), the hazard ratio (HR) is calculated by exponentiating the beta for vaccination. For calculations that apply to the post-Omicron time range, the HR is calculated by summing the vaccination and interaction term betas and exponentiating the result.

Surveillance testing for those fully vaccinated was required less frequently, and performed once a month rather than every other week (Figure S2, Figure S3). To account for potential bias, we conducted the analysis at a monthly level with an overall result for the month. If a participant with biweekly testing had any positive test in a month, they were classified as positive for that month. By structuring the data this way, participants with more frequent testing were not overrepresented compared to those tested less often due to vaccination.

Participants missing key variables necessary for the analysis (age, gender, or AGP for the sub-analysis) were excluded. Few participants were missing age and gender information (8/410 or 2% of participants and 29/8,243 or 0.4% observations). One individual did not provide a response to the frequency of AGP, which accounted for 1/116 or less than 1% of the survey subset.

To adjust for potential informative censoring, we included propensity score weighting in our main model to adjust for the probability of censoring (Figure S4). A participant was considered censored if they had not tested positive at the time of their final observation. Weights were created by predicting likelihood of not dropping out with the *WeightIt* package in R and propensity score weighting using generalized boosted modeling (“gbm”), stabilized weights, and a standardized mean differences target of 0.05^20^.

Analyses were conducted in R version 4.4.0^21^.

#### Supplementary

The number of covariates available in this dataset was limited. As there may be additional, unmeasured confounding, a sensitivity analysis was conducted: we assessed the strength required of an unknown confounder to reverse the direction of an estimate using the E-Value method^22^.

## Results

There were 8,270 tests after restricting to the first positive test for each participant or end-of-follow-up (Table 1). As expected, faculty had the highest average age (59 years), while students had the lowest (28 years). Most faculty and students were male (73%, 221/304), while the staff group were predominantly female (85%, 83/106). More than half of the population (67%, 276/410) had received two or more vaccines by the end of follow-up. On average, participants were followed for 56 weeks.

**Table 1:** Demographics and testing characteristics of dental school participants who underwent SARS-CoV-2 surveillance testing, including repeat positive tests. Survival analyses were restricted to each participant’s first positive test.

Characteristic	Faculty *N* = 104^*1*^	Staff *N* = 106^*1*^	Student *N* = 200^*1*^	Overall *N* = 410^*1*^
Age				
Median (Q1, Q3)	62 (48, 68)	36 (29, 45)	27 (26, 29)	31 (27, 48)
Mean (SD)	59 (13)	38 (11)	28 (3)	38 (16)
Min, Max	34, 85	19, 69	22, 43	19, 85
Unknown	0	8	0	8
Gender				
F	15 (14%)	83 (85%)	68 (34%)	166 (41%)
M	89 (86%)	15 (15%)	132 (66%)	236 (59%)
Unknown	0	8	0	8
Total # of Tests				
Median (Q1, Q3)	22 (12, 30)	21 (8, 32)	25 (21, 28)	24 (15, 29)
Mean (SD)	22 (12)	20 (13)	24 (6)	22 (10)
Min, Max	1, 50	1, 45	10, 41	1, 50
Total Positive Tests				
0	85 (82%)	68 (64%)	122 (61%)	275 (67%)
1	16 (15%)	32 (30%)	64 (32%)	112 (27%)
2	3 (2.9%)	6 (5.7%)	14 (7.0%)	23 (5.6%)
Weeks from first observation to first positive or last observation				
Median (Q1, Q3)	73 (33, 85)	54 (20, 85)	63 (50, 77)	62 (33, 78)
Mean (SD)	59 (29)	51 (33)	57 (24)	56 (28)
Min, Max	0, 91	0, 92	0, 92	0, 92
Max Vaccines Reported by end of follow-up				
0	25 (24%)	39 (37%)	52 (26%)	116 (28%)
1	4 (3.8%)	8 (7.5%)	6 (3.0%)	18 (4.4%)
2	64 (62%)	52 (49%)	126 (63%)	242 (59%)
3	11 (11%)	7 (6.6%)	16 (8.0%)	34 (8.3%)
Vaccine Brand				
J & J	0 (0%)	2 (1.9%)	1 (0.5%)	3 (0.7%)
Moderna	21 (20%)	7 (6.6%)	6 (3.0%)	34 (8.3%)
Pfizer	58 (56%)	58 (55%)	141 (71%)	257 (63%)
Unvaccinated	25 (24%)	39 (37%)	52 (26%)	116 (28%)

^*1*^n (%).

**Fig. 1:**
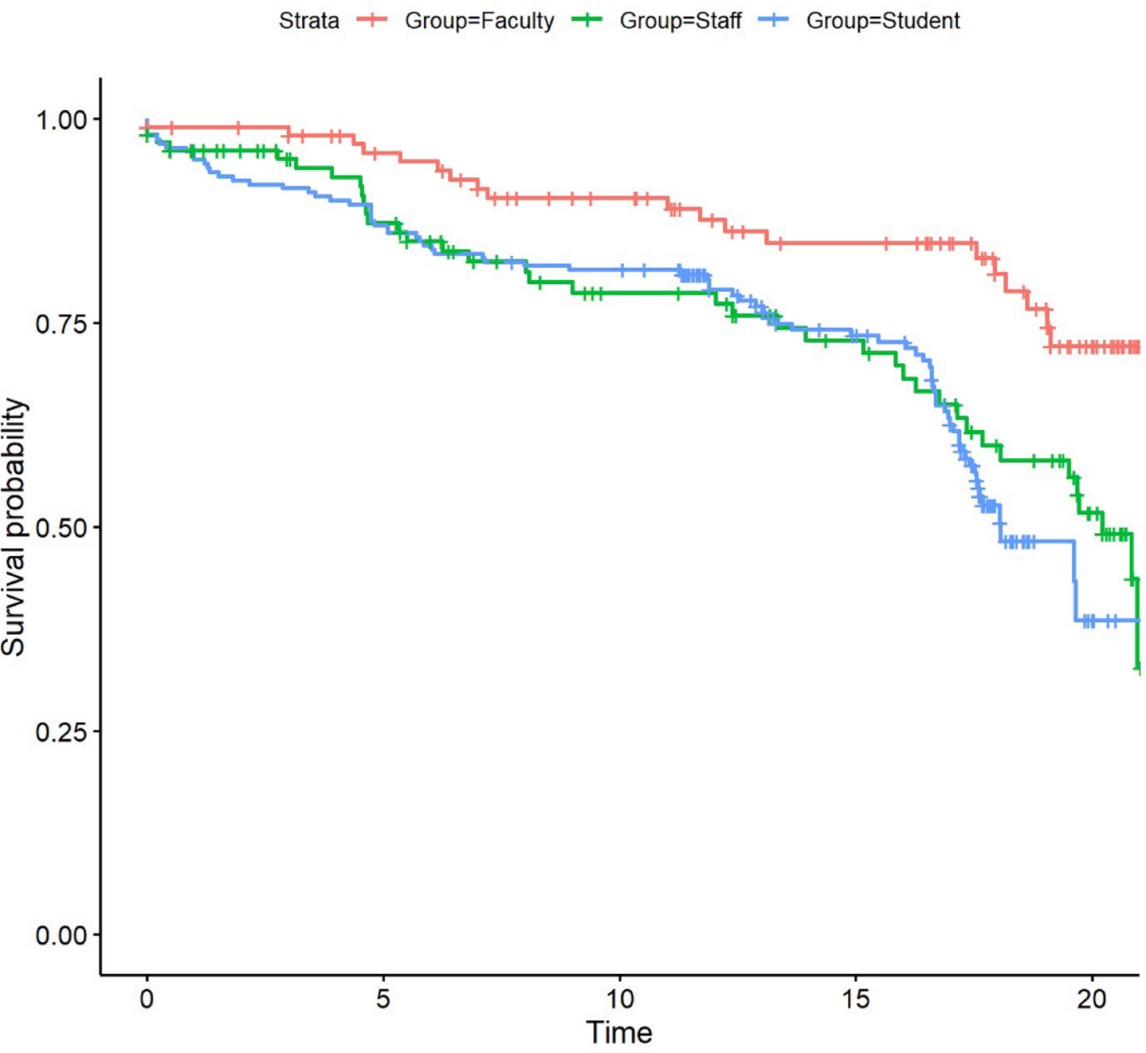
Survival probability (Kaplan Meier Curve) of participants for positive COVID-19 screening test within dental school faculty, staff, and students. Kaplan–Meier curves are shown as an unadjusted descriptive visualization of time to first positive SARS-CoV-2 PCR test by work role. Time is in months since study entry, and vertical tick marks represent censoring.

**Fig. 2:**
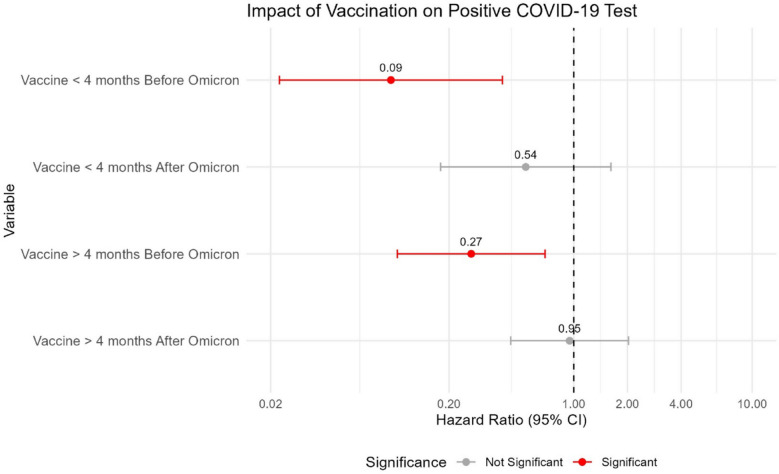
Risk of positive COVID-19 surveillance test among dental faculty, staff, and students at various vaccination states. Model: Cox Proportional Hazards model with time-dependent covariates. The original weekly data has been transformed to a monthly level for this model. Includes 4640 tests from 399 participants. Numbers shown are hazard ratios with a 95% confidence interval on a log scale.

There were 158 positive tests, with 60 (38%) occurring in January 2022. Of the original 410 participants in the cohort, 33% (135/410) tested positive at least once during the study period. As the analysis was restricted to the first positive and those who had not been vaccinated prior to their first test, only 133 positive tests were included in the regression. The crude incidence of participants testing positive for SARS-CoV-2 pre- and post- the arrival of the omicron variant was 16.3 (76 pre-Omicron new cases/4,675 person*months) and 200.0 (57 post-Omicron new cases/285 person*months) per 1,000 person-months, respectively. Informal case investigations conducted as part of routine follow-up suggested that many participants who tested positive reported exposures in the home or social settings outside of the workplace. Figure 1 indicates the survival probability of participants for a positive screening test for SARS-CoV-2 using a Kaplan-Meier curve. Our main analysis (Table 2; Fig. 2) found Omicron to have a significant impact on vaccination effect when the most recent receipt of vaccination was greater than 4 months. Vaccine effectiveness on susceptibility of infection declined significantly over time and with the emergence of the Omicron variant. Prior to Omicron, effectiveness was estimated at 91% (HR 0.09, 0.02–0.40) when vaccination had occurred within the previous four months. After Omicron became dominant, effectiveness dropped to 46% (HR 0.54, CI 0.18–1.62) among individuals whose last dose was less than four months earlier.

**Table 2:** Assessment of interaction between vaccination and Omicron time period. Model: Cox Proportional Hazards Regression with time dependent covariates with weighted to adjust for dropout. The original weekly data has been transformed to a monthly level for this model. Includes 4640 tests from 399 participants.

Characteristic	log(HR)^1^	95% CI^1^	*p*-value
Vaccination Status			
Unvaccinated or not fully vaccinated	—	—	
Vaccinated < 4 months	-2.4	-3.8, -0.92	**0.001**
Vaccinated > 4 months	-1.3	-2.3, -0.37	**0.006**
Omicron			
Pre-Omicron Surge	—	—	
Omicron Surge	1.2	0.07, 2.4	**0.038**
Age	-0.01	-0.04, 0.01	0.2
Gender			
F	—	—	
M	-0.10	-0.49, 0.29	0.6
Group			
Faculty	—	—	
Staff	0.61	-0.06, 1.3	0.076
Student	0.65	-0.09, 1.4	0.085
Vaccination Status * Omicron			
Vaccinated < 4 months * Omicron Surge	1.7	-0.06, 3.5	0.059
Vaccinated > 4 months * Omicron Surge	1.3	0.07, 2.5	**0.038**

^*1*^HR = Hazard Ratio, CI = Confidence Interval.

Additional analysis was performed on a subgroup of the population for which survey data of exposure to AGP was available (Table S2). There were 115 participants who completed a survey and responded to the frequency they were exposed to AGP during the observation period. These participants had an average of 27 (SD 7, range 8–50) tests per person and 2,750 total tests. The majority of volunteer participants who completed the survey were actively involved in direct patient care and frequently performed aerosol-generating procedures (AGPs). Because this group was larger than those with less frequent AGP involvement, frequent AGP exposure (once a week or more) was used as the reference category to improve the precision of the estimates. The frequency of involvement with AGP (once a month or less vs. once per week or more) was not found to be a statistically significant predictor of a positive test (Table 3). However, given the limited sample size for AGP data, these findings should be considered exploratory and hypothesis-generating.

**Table 3:** Effect of self-reported aerosol generating procedure frequency on positive COVID-19 test in dental school faculty, staff, and students. Model has been adjusted for dropout using weights through generalized boosted modeling (“gbm”) with stabilized weights. Model: Cox Proportional Hazards Regression with time dependent covariates. The original weekly data has been transformed to a monthly level for this model. Includes 1503 tests from 115 participants.

Characteristic	HR^1^	95% CI^1^	*p*-value
Frequency of Aerosol Procedures (Condensed)			
Often/Frequently (Once per week or more)	—	—	
Once a month or less	2.31	0.81, 6.56	0.12
Vaccination Status			
Unvaccinated or not fully vaccinated	—	—	
Vaccinated < 4 months	0.07	0.02, 0.23	**< 0.001**
Vaccinated > 4 months	0.13	0.05, 0.36	**< 0.001**
Age	0.97	0.93, 1.00	0.062
Gender			
F	—	—	
M	0.76	0.36, 1.62	0.5
Group			
Faculty	—	—	
Staff	0.49	0.12, 1.99	0.3
Student	1.21	0.29, 5.08	0.8

^*1*^HR = Hazard Ratio, CI = Confidence Interval.

The effect sizes needed for a potential confounder to reverse the direction of any of our significant estimates can be seen in Table S3.

## Discussion

The COVID-19 pandemic presented significant challenges in the delivery of healthcare, particularly when involving AGP. The interventions employed at the UUSOD for the continuity of dental care include a variety of protective measures, such as modifications to the environment, regular dental care worker screening, and vaccination. As ongoing surveillance testing for SARS-CoV-2 was available in DCWs, we were able to assess risk to the dental team. Different work roles had different levels of exposure to AGP and we found no significant difference between work groups and the risk of testing positive for SARS-CoV-2 after weighting for dropout, and accounting for vaccination, age, and gender. Additionally, we found no significant relationship between the reported frequency of involvement with AGP and the risk of a positive test.

Previous research has repeatedly shown that infections among healthcare workers are primarily driven by community exposure^23–26^. For example, viral sequencing of SARS-CoV-2 in US healthcare workers found most infections to be genetically similar to those circulating in the community and could not usually be linked to a patient or coworker^23^. Another more recent study found exposure to illness outside of work strongly predictive of positive tests, while exposure at work was protective^26^. Consistent with this literature, informal case investigations conducted during this study suggested that many participants reported exposures in the home or social settings, although these observations were not a formal analysis. Other research has reported dental practitioners to have low rates of COVID-19^12,13,27,28^. This may be due to the use of preventive measures like the ones reported here, which seems to have been common among DCWs. For example, preventive measures were almost universally reported early in the pandemic in a study of dental hygienists^10^. Our study compared work roles in the dental school with differing exposure to patients and procedures. However, we lack data on risk variation in other healthcare roles. Comparative studies of COVID-19 infection risk across healthcare professions are scarce, especially in dentistry.

Our vaccination effect estimates on infection susceptibility in DCWs are consistent with findings from other published research. Our E-value analysis indicates that moderate to strong unmeasured confounding would be required to explain away the observed associations, although residual confounding cannot be excluded. Prior to the emergence of the Omicron variant, studies estimated the first SARS-CoV-2 mRNA vaccines to have higher effectiveness in real-world settings^29,30^, with estimates near or above 90%. However, effectiveness decreased dramatically after the emergence of the Omicron variant^31^. This reduction is likely due to waning immunity and Omicron’s spike protein mutations, which reduce antibody binding and neutralization. Despite these changes, work role was not associated with increased risk after adjusting for vaccination status, age, and gender, suggesting that community transmission and immune escape were more influential than occupational exposure. Our findings indicate that differences in professional roles and reported exposure to aerosol-generating procedures (AGPs) did not significantly influence infection risk after adjusting for vaccination status, age, and gender. This suggests that preventive measures, such as consistent use of high-filtration respirators (e.g., N95 or KN95), environmental controls, and adherence to infection control protocols, effectively minimized occupational transmission, even among those with frequent AGP involvement. These results align with prior research showing that PPE use and strict hygiene habits are critical for reducing risk among healthcare workers^32^. Clinically, maintaining rigorous PPE standards and reinforcing vaccination remain essential, not only for SARS-CoV-2 but also for other respiratory pathogens that spread via aerosols, such as influenza and RSV. Continued emphasis on booster doses, proper respirator fit, and ventilation improvements will help sustain protection in dental and other healthcare settings.

Maintaining up-to-date vaccination through booster doses and vaccinations updated for new variants improves protection^33,34^. While vaccination does appear to be less effective at prevention of infection in the post-Omicron era, it is important to note that estimates of vaccination effectiveness against severe illness and death remain high^35,36^. Additionally, the intensity of the pandemic changed drastically during the Omicron era, as evidenced by the large difference between incidence rates before and after the variant’s emergence. As the Omicron variant spans over a much smaller section of our study and fewer individuals may have been vaccinated within 4 months at that time, we are also limited in our ability to detect significant differences in this category.

### Limitations

Several factors limit this study. Individuals may not have reported a positive test result if testing was performed outside the surveillance program. Our estimate and inference of AGP impact on COVID-19 positive surveillance tests is limited due to sample size and may have been underpowered to detect modest associations. As such, the absence of a statistically significant association between AGP frequency and risk of positive test should not be interpreted as evidence of no effect. Rather, these findings should be considered exploratory and hypothesis-generating. Additionally, while the subset of survey data was collected during the overall surveillance study, the surveillance study ran for a longer period, and the survey dates did not match the surveillance dates. The survey has far fewer time points than the surveillance study. We assume that AGP exposure does not change much from the few survey time points available and that exposure does not change during the surveillance study. Additionally, AGP exposure was based on self-reported frequency of involvement, which does not allow us to determine the precise level of aerosol exposure experienced by individuals. A key limitation of this study is that AGP exposure was assessed using a survey item not specifically tailored to dental practice. The examples provided in the survey reflect medical procedures and do not explicitly include common dental AGPs such as ultrasonic scaling or high-speed handpiece use. Although participants likely interpreted the question within the context of their clinical roles, this measure may not fully capture the nuances of aerosol exposure in dental settings. Therefore, findings related to AGP exposure should be interpreted as reflecting a proxy measure rather than a precise assessment of dental-specific AGPs. Future studies may benefit from incorporating simulation-based modeling to better quantify aerosol exposure when epidemiological data is unavailable.

This information may not reflect all dental schools or clinical settings if COVID-19 mitigation practices and facilities differ. The findings may not reflect other respiratory diseases as only Sars-CoV-2 was tested. In this study, we use PCR surveillance testing to evaluate infection, but we do not include serology data, such as IgG antibody levels. As our design focused on observed infection risk based on PCR testing and not IgG or other immunologic correlates, we were unable to evaluate individual immune response following vaccination. PCR surveillance can also only detect active infections, though some individuals may continue to show PCR positivity for two weeks or more^37,38^. As such, individuals who experienced asymptomatic or unreported infections between testing intervals may not have been captured and could result in an underestimation of total infections. Finally, while we suspect positive tests to be primarily driven by community exposure, we did not directly measure community activity. We employed several methods to handle potential bias in our population, including condensing the study timescale and utilizing propensity score weighting for dropout.

## Conclusion

More than a third of all positive tests during the 22-month study occurred during one month of the Omicron wave. This sudden increase in positive tests was not observed in previous surges and demonstrates the intensity of the Omicron wave. We found high vaccine effectiveness prior to the Omicron surge (91%, HR 0.09, 0.02–0.40), which decreased after the emergence of Omicron (46%, HR 0.54, CI 0.18–1.62). Additionally, we did not find a significant difference between patient-facing groups who had different reported AGP exposures or work roles which have different exposures to patients. Our results support encouraging DCWs to remain up to date on vaccination and continue engaging in preventive measures that protect them, regardless of their work role.

## Supplementary Information

Below is the link to the electronic supplementary material.

Supplementary Material 1

## Data Availability

The data that support the findings of this study are not openly available due to reasons of study participant privacy. Data are available from the authors upon reasonable request and with permission from the institutional review board of the University of Utah. For additional information on data availability, please contact Matthew Samore at matthew.samore@hsc.utah.edu.
