# Supplementary material for: SARS-CoV-2 surveillance and vaccine effectiveness among dental healthcare workers during the omicron surge
Source: Sci Rep. 2026 May 5;16:20633. doi: 10.1038/s41598-026-50216-8 (PMC13333859; doi:10.1038/s41598-026-50216-8)
Supplement: Supplementary file 1 — Supplementary Material 1 [file 41598_2026_50216_MOESM1_ESM.pdf]

Supplemental

Title: Vaccination, Work Practices, and Aerosol Generating Procedures in SARS-CoV-2 Surveillance Outcomes Among Dental Healthcare Workers

Contents

Tables 2

Table S1, Mitigation Timing 2

Table S2, Subgroup Demographics 3

E Value 4

Table S3, E Value 4

Figures 5

Figure S1, Selection Diagram 5

Figure S2, Time Between Tests (Overall) 6

Figure S3, Time Between Tests (Group) 7

Figure S4, Balancing Figure 8

Tables

Table S1, Mitigation Timing

List of COVID-19 mitigation efforts and timing for the UUSOD

	2020								2021												2022	
Policy	May	Jun	Jul	Aug	Sep	Oct	Nov	Dec	Jan	Feb	Mar	Apr	May	Jun	Jul	Aug	Sep	Oct	Nov	Dec	Jan	Feb
Surveillance Testing (every two weeks for all)	X	X	X	X	X	X	X	X	X	X	X	X										
Surveillance Testing (monthly for vaccination, or every two weeks for unvaccinated)													X	X	X	X	X	X	X	X	X	X
Patient pre-appointment testing	X	X	X	X	X	X	X	X	X	X	X	X	X									
Increased Clinic Space	X	X	X																			
Added HEPA filters	X	X	X	X	X	X	X	X	X	X	X	X	X	X	X	X	X	X	X	X	X	X
Reduced Aerosolized procedures	X	X	X																			
Required respirator for all clinical providers during patient care	X	X	X	X	X	X	X	X	X	X	X	X	X	X	X	X	X	X	X	X	X	X

Table S2, Subgroup Demographics

Subgroup Population Table

**Characteristic**	**Faculty** N = 24[1]	**Staff** N = 23[1]	**Student** N = 69[1]	**Overall** N = 116[1]
Age				
Median (Q1, Q3)	66 (50, 74)	39 (33, 55)	28 (26, 30)	31 (27, 48)
Mean (SD)	62 (14)	44 (13)	29 (4)	39 (16)
Min, Max	38, 85	24, 69	22, 43	22, 85
Gender				
F	5 (21%)	20 (87%)	30 (43%)	55 (47%)
M	19 (79%)	3 (13%)	39 (57%)	61 (53%)
Total # of Tests				
Median (Q1, Q3)	30 (27, 38)	29 (22, 36)	26 (22, 28)	27 (23, 30)
Mean (SD)	33 (8)	28 (9)	25 (5)	27 (7)
Min, Max	23, 50	8, 40	12, 37	8, 50
Total Positive Tests				
0	18 (75%)	13 (57%)	42 (61%)	73 (63%)
1	5 (21%)	6 (26%)	22 (32%)	33 (28%)
2	1 (4.2%)	4 (17%)	5 (7.2%)	10 (8.6%)
Frequency of AGP[2]				
Never/Rarely	4 (17%)	10 (43%)	9 (13%)	23 (20%)
Occasionally (at least once per month)	3 (13%)	4 (17%)	1 (1.4%)	8 (7.0%)
Often (at least once per week)	5 (22%)	0 (0%)	11 (16%)	16 (14%)
Frequently (at least once a shift)	11 (48%)	9 (39%)	48 (70%)	68 (59%)
Unknown	1	0	0	1
Frequency of AGP[2] (Condensed)				
Often/Frequently (Once per week or more)	16 (70%)	9 (39%)	59 (86%)	84 (73%)
Once a month or less	7 (30%)	14 (61%)	10 (14%)	31 (27%)
Unknown	1	0	0	1
Weeks from first observation to first positive or last observation				
Median (Q1, Q3)	86 (78, 90)	85 (26, 90)	66 (49, 77)	74 (49, 83)
Mean (SD)	77 (21)	62 (31)	57 (23)	62 (26)
Min, Max	27, 91	12, 92	0, 88	0, 92
Max Vaccines Reported by end of follow-up				
0	2 (8.3%)	7 (30%)	19 (28%)	28 (24%)
1	2 (8.3%)	1 (4.3%)	1 (1.4%)	4 (3.4%)
2	14 (58%)	12 (52%)	44 (64%)	70 (60%)
3	6 (25%)	3 (13%)	5 (7.2%)	14 (12%)
Vaccine Brand				
Moderna	3 (13%)	0 (0%)	1 (1.4%)	4 (3.4%)
Pfizer	19 (79%)	16 (70%)	49 (71%)	84 (72%)
Unvaccinated	2 (8.3%)	7 (30%)	19 (28%)	28 (24%)

[1]n (%) [2]Aerosol-generating procedures

E Value

Table S3, E Value

E Values of significant estimates from all models assessing likelihood of positive COVID-19 surveillance test. **Showing only statistically significant estimates (95% CI excludes 1).**

Model	Variable	HR	Lower 95% CI	Upper 95% CI	E-Value
AGP Model	Vaccinated < 4 months	0.07	0.02	0.23	10.99
AGP Model	Vaccinated > 4 months	0.13	0.05	0.36	7.04
Vaccine Omicron Interaction Model	Vaccinated < 4 months	0.09	0.02	0.40	8.80
Vaccine Omicron Interaction Model	Vaccinated > 4 months	0.27	0.10	0.69	4.35
Vaccine Omicron Interaction Model	Omicron Surge	3.45	1.07	11.14	4.08
Vaccine Omicron Interaction Model	Vaccinated > 4 months: Omicron Surge	3.56	1.07	11.85	4.18

Figures

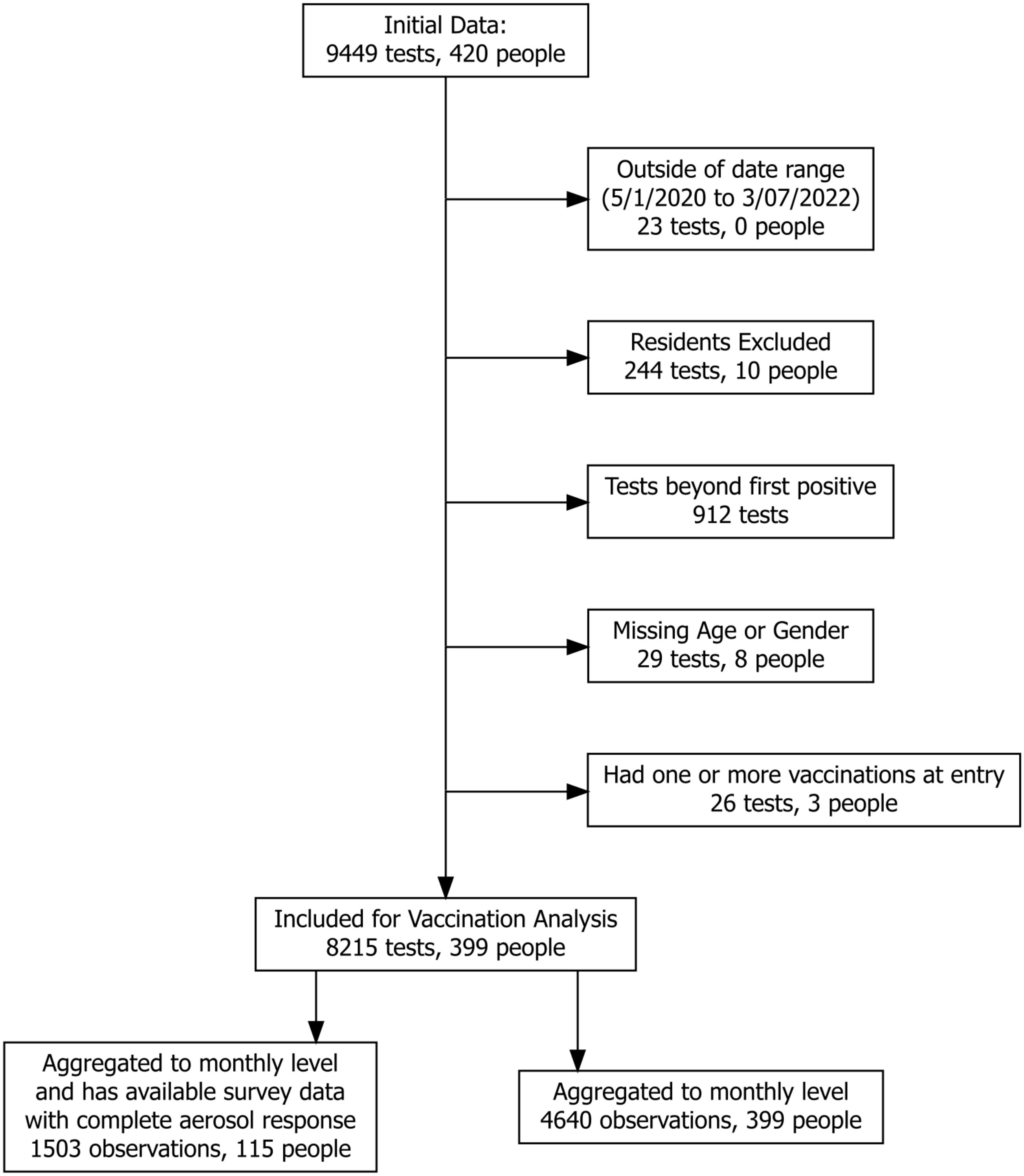

Figure S1, Selection Diagram

Selection diagram for analysis of dental school surveillance tests

Average Time Between Tests (Overall)

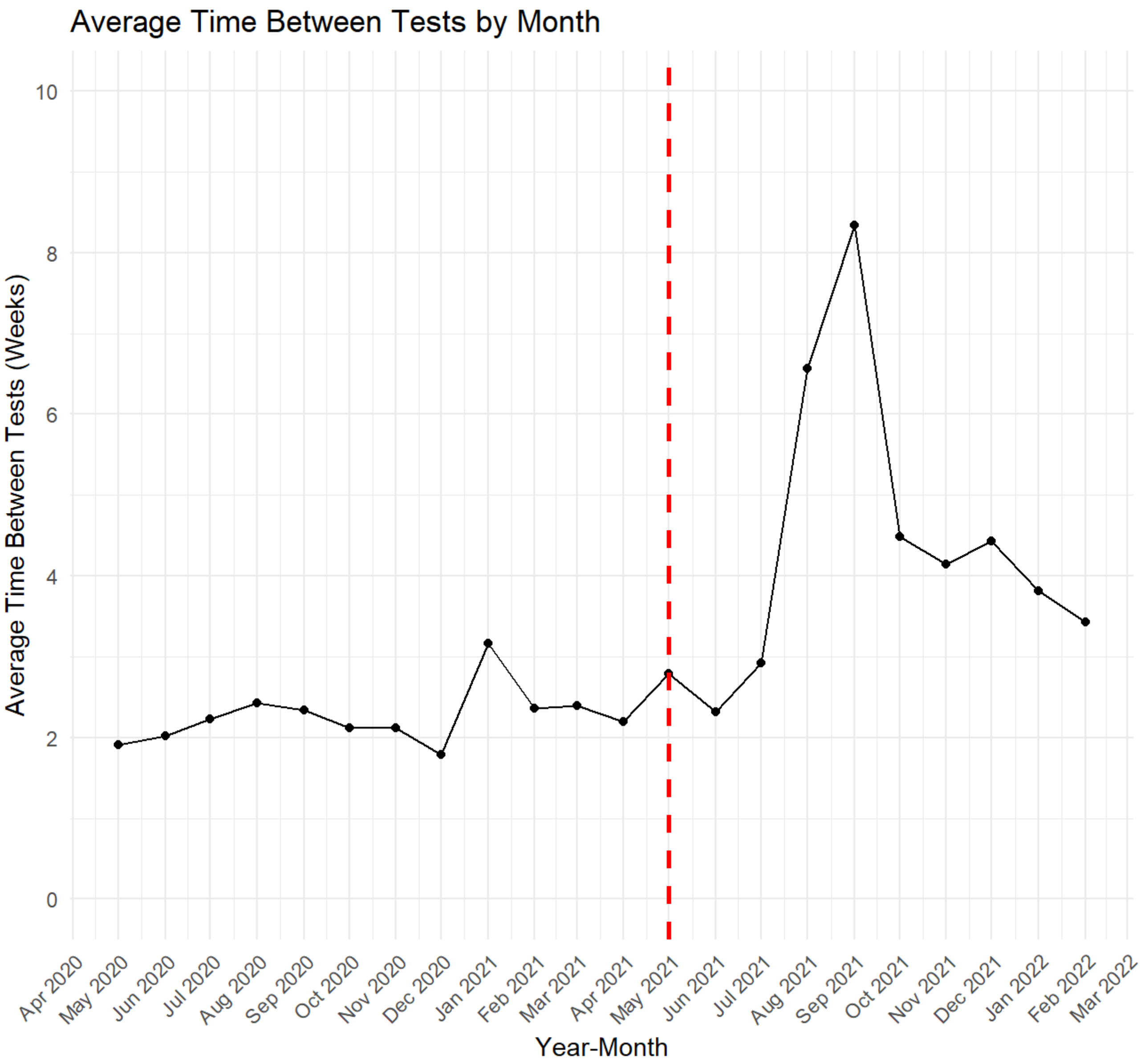

Figure S2, Time Between Tests (Overall)

Average time between SARS-CoV-2 screening tests in weeks. The dashed red vertical line represents the time at which the school of dentistry implemented a policy that allowed vaccinated individuals to be tested once a month rather than every other week.

Average Time Between Tests (By Group)

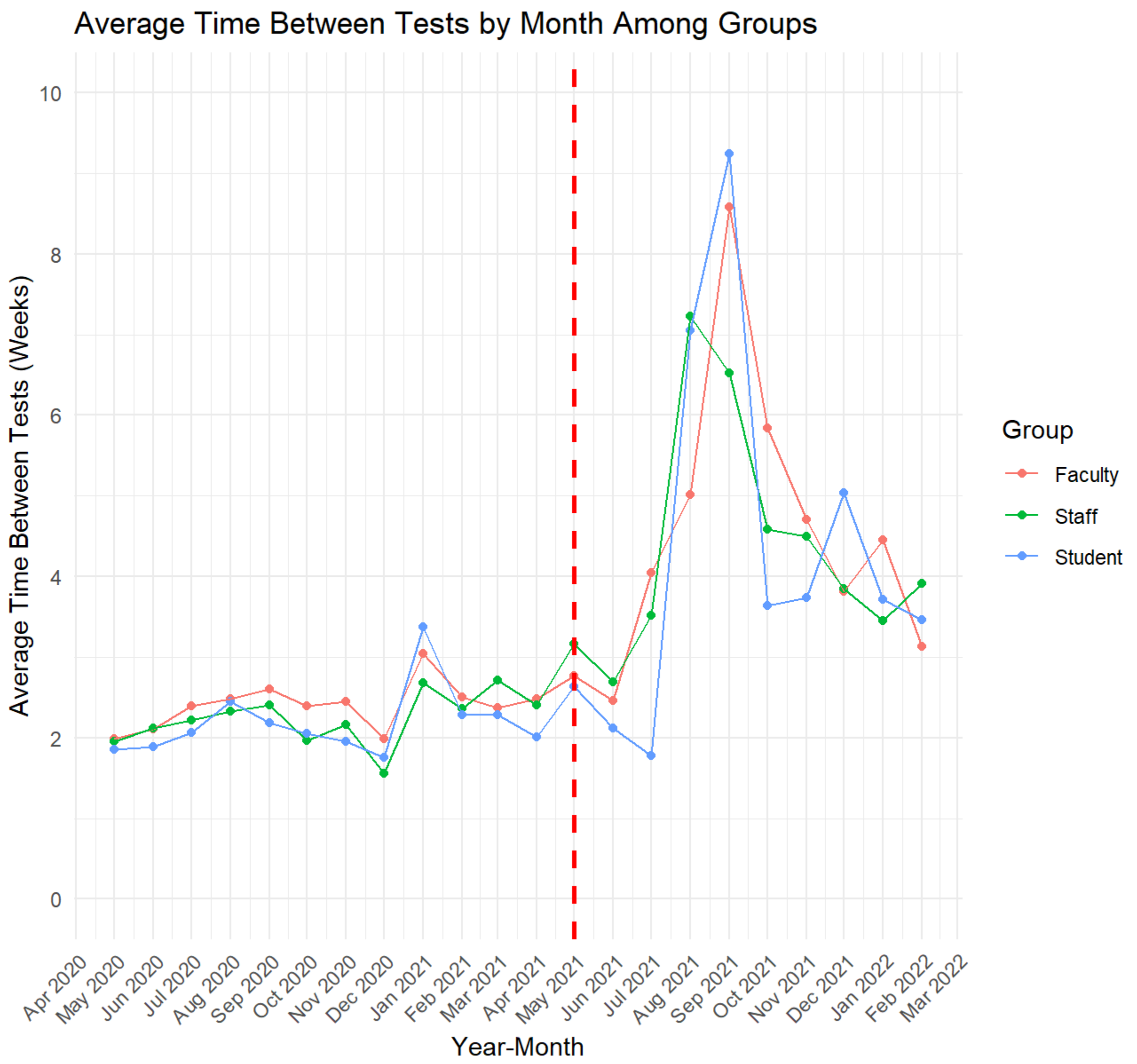

Figure S3, Time Between Tests (Group)

Average time between SARS-CoV-2 PCR screening tests in weeks among Faculty, Staff, and Students. The dashed red vertical line represents the time at which the school of dentistry implemented a policy that allowed vaccinated individuals to be tested once a month rather than every other week.

Dropout Balancing Table

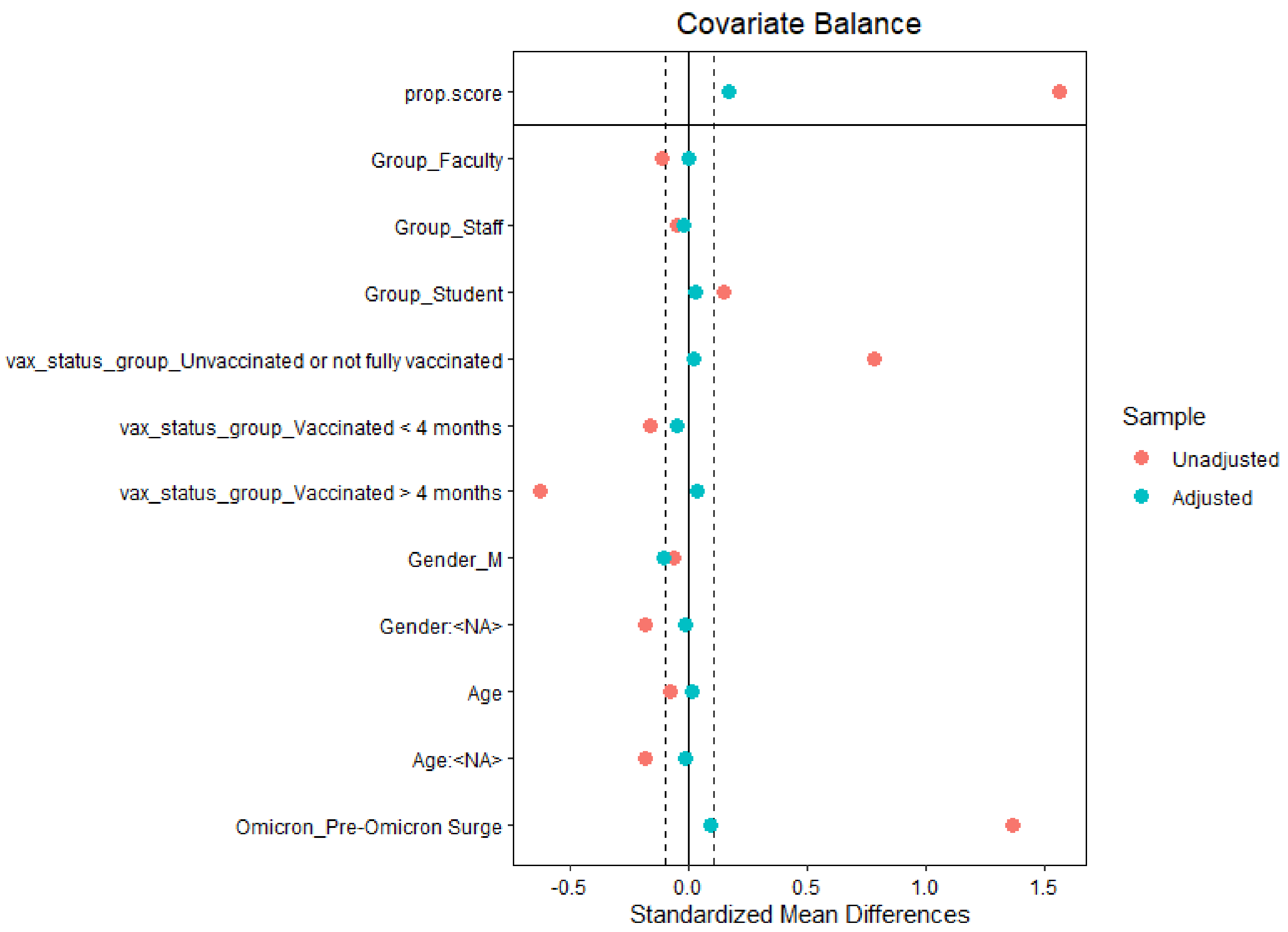

Figure S4, Balancing Figure

Covariate Balance of adjusted and unadjusted variables. Participants who dropped out of the survey before the final month and had not already tested positive were considered as having dropped out. Generalized boosted modeling ("gbm") with stabilization was used to create weights. Weights were trimmed at the 99.9th percentile, and 0.1 as a threshold for a balanced covariate (represented here by a dotted line).
